# A practical guide for investigating cardiac physiology using living myocardial slices

**DOI:** 10.1007/s00395-020-00822-y

**Published:** 2020-09-10

**Authors:** S. A. Watson, A. Dendorfer, T. Thum, F. Perbellini

**Affiliations:** 1 Institute of Molecular and Translational Therapeutic Strategies (IMTTS), Hannover Medical School, Hanover, Germany; 2 National Heart and Lung Institute, Imperial College London, London, UK; 3 King’s College Hospital, London, UK; 4 Walter-Brendel-Centre of Experimental Medicine, University Hospital, LMU Munich, Munich, Germany

**Keywords:** Cardiovascular models, Multicellularity, Living myocardial slice, Translational research

## Abstract

Ex vivo multicellular preparations are essential tools to study tissue physiology. Among them, the recent methodological and technological developments in living myocardial slices (LMS) are attracting increasing interest by the cardiac research field. Despite this, this research model remains poorly perceived and utilized by most research laboratories. Here, we provide a practical guide on how to use LMS to interrogate multiple aspects of cardiac function, structure and biochemistry. We discuss issues that should be considered to conduct successful experiments, including experimental design, sample preparation, data collection and analysis. We describe how laboratory setups can be adapted to accommodate and interrogate this multicellular research model. These adaptations can often be achieved at a reasonable cost with off-the-shelf components and operated reliably using well-established protocols and freely available software, which is essential to broaden the utilization of this method. We will also highlight how current measurements can be improved to further enhance data quality and reliability to ensure inter-laboratory reproducibility. Finally, we summarize the most promising biomedical applications and envision how living myocardial slices can lead to further breakthroughs.

## Introduction: why use living myocardial slices?

Our collective understanding of the cellular processes involved in cardiac physiology and remodeling is primarily derived from isolated cells, where complex tissue architecture, extracellular matrix, native multicellularity and intercellular communication are lost. While the simplicity of these platforms is attractive, the data obtained are somewhat ‘oversimplified’ and cells cultured in vitro tend to display aberrant behaviors and altered phenotype [2, 11]. Knowledge has also been derived from whole heart and isolated muscle (papillary and trabeculae) studies, which both retain maximal proximity to the in vivo environment. However, these models lack significant throughput and their use for chronic in vitro studies is limited [38, 45].

What is needed are novel multicellular models, capable of better simulating the physiological milieu, that bridge the gap between conventional cultures and complex in vivo preparations, while facilitating the incorporation of findings within the wider spectrum of cardiovascular research. Living myocardial slices (LMS) provide a novel platform to study new aspects of cardiac biology, which, due to advancements in preparation methodology and in vitro culture, are attracting increasing interest from both the academic and commercial arms of cardiovascular research [45].

**Fig. 1:**
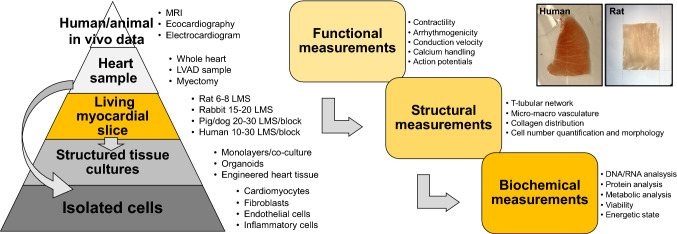
Living myocardial slices within the cardiac research pipeline. LMS can be prepared from heart samples, including whole heart animal models and human myectomy or LVAD samples. Interrogation of cardiac function, structure and biochemistry can then be performed on LMS. It is also possible to isolate individual cardiac cell populations from LMS. All of the data obtained can then be integrated with human/in vivo data the LMS are derived from

The objective of this review is to provide a guide for those interested in, but not sufficiently familiar with, the technical aspects associated with LMS. Here, we present a simplified “pyramid model” overview (Fig. 1) of the various experiments that can be performed, ranging from the macroscopic, tissue-level assessment of structure and function to single-cell RNA experiments. We provide basic instruction on LMS preparation and assessment, including practical tips on experimental techniques. We briefly compare LMS to other ex vivo techniques and discuss the different animal models they can be prepared from. Our hope is that this review can serve as a preface to LMS for non-experts, providing an open door for further investigation of the vast array of techniques that can be utilized and prompting application of LMS among various specialist laboratories across the cardiovascular research community.

## Integration of LMS in the cardiac research pipeline

Various animal models have been developed to reproduce cardiac pathophysiological conditions [6] and their utilization has led to substantial improvements in our understanding of disease pathogenesis, diagnosis and both preventative and therapeutic interventions. In the cardiovascular field, methodologies such as electrocardiograms, echocardiography and magnetic resonance imaging provide scientists with multiple data to assess cardiac function [5]. Following in vivo data acquisition, animals are killed and tissue samples collected for histological and molecular analysis. LMS fit between these two research stratifications. LMS can be prepared from freshly collected specimens and used to acquire cellular and subcellular parameters in the context of the cardiac multicellular environment. The functional, structural, biochemical and genomic data acquired could then be integrated with existing in vivo and in vitro data, with the potential to provide a more comprehensive and integrated understanding. Extensive comparison of the advantages and disadvantages of LMS and other multicellular cardiac preparations have already been published [38].

## LMS preparation

LMS preparation was first described in the 1930s, when tissue slices were prepared by manual dissection [34, 46]. In the 1990s, two essential steps to enhance LMS viability and functionality were introduced. The first was the automation and standardization of the cutting process using high-precision vibratomes. The second was the slicing of tissue blocks in the epicardium-tangential plane, which ensures optimal alignment of cardiomyocytes with the slicing blade and results in substantially reduced cell damage [31, 47]. LMS are now prepared from almost all of the animal species including mice [20, 39, 40, 52], rats [4–7, 11, 29, 35, 39 49, 50, 53], guinea pigs [6, 7, 48], rabbits [48], dogs [8, 38, 39] and pigs [36]). LMS can also be prepared from human specimens [3, 9, 29, 41, 43, 44] providing great potential for translational studies. Preparation of LMS is straightforward when using healthy tissue, but several aspects must be considered when using pathological models. LMS preparation is likely to be easily applied to animal models of cardiac hypertrophy, such as TAC or hypertensive rats, where macroscopic tissue architecture is preserved. However, models of extensive fibrosis, such as myocardial infarction, provide a challenge. The presence of extensive fibrotic regions occasionally impedes smooth cutting and inevitably adds a source of inhomogeneity. However, slicing can still be achieved by slowing the blade advancement speed and increasing vibration amplitude. Accelerated degeneration of cultured LMS from human ischaemic cardiomyopathy samples seems to indicate that general pathophysiological features of fibrotic myocardium are maintained within LMS [26].

### Heart isolation/specimen collection

Appropriate specimen collection and storage is the primary and most crucial step. For small animal models, rapid extraction and cooling of the heart are sufficient to prevent cardiac tissue injury. For large animals, in situ cardioplegic arrest is highly recommended, as immersion of large portions of the myocardium in cold solution may not facilitate sufficiently fast cooling, resulting in tissue damage. Cold storage and rapid transfer to the laboratory are recommended, typically in a high [K^+^], low [Ca^2+^] solution with an excitation–contraction uncoupler (2,3-butanedione monoxime) [3, 9, 29, 41, 43, 44]. A thorough characterization of different cooling methodologies and storage solutions is yet to be conducted, but would be useful to further optimize this step and minimize tissue damage. For specimens of explanted human hearts, a convenient approach of rapid cooling and cardioplegic hypothermic (4 °C) preservation has been shown to permit storage for > 30 h without impairment of LMS functionality. This enables the exchange of tissue samples between remote clinics and laboratories by standard courier services [9].

### Preparation

LMS are prepared with a high precision vibratome. Following identification of cardiomyocyte orientation and careful tissue block positioning (with the epicardium facing the specimen holder), the vibration of the blade detaches (rather than transects) neighboring cardiomyocytes [44]. Cardiomyocytes are large, distinct cells, connected to their neighbors by gap junctions. Thus, any damage incurred during preparation is limited to individual cells by this architectural arrangement. This results in approximately 40–60% viable cardiomyocytes on the LMS surface, as demonstrated with confocal microscopy [44] or calcium imaging [43]. Alternative faster and cheaper machines are available, but these tend to lack control of blade vibration in the *z*-axis, which must be kept to < 1 µm. Currently, the most popular brands for high-precision vibratomes are Leica and Camden Instruments. Considering that both machines allow precise control of blade vibration, a very similar outcome is expected; however, a direct comparison in LMS quality and functionality has not been done yet. To maintain oxygen diffusion to all of the cells within LMS, a thickness ~ 300 µm is recommended. This results in approximately 11–13 layers of cardiomyocytes, with total estimated cell viability ~ 95% [44]. For a step-by-step protocol for LMS preparation, we re-direct the reader to [44]. When optimally prepared, LMS do not demonstrate spontaneous beating activity and will only contract if electrically stimulated. Spontaneous or arrhythmic events are an indicator of tissue damage and LMS preparation should be reviewed. LMS are a medium-throughput research model, with several LMS generated from one heart/specimen. Approximately, three LMS can be prepared from a mouse heart, however, LMS number rises significantly when larger animal models are used. One can generate 6–8 LMS from a rat left ventricle (LV) and approximately 15–20 from a rabbit LV. Tissue blocks (1 cm^3^) prepared from larger animals, such as dog, pigs and humans, can produce 20–30 LMS. Considering several tissue blocks can be prepared from each specimen, hundreds of LMS can be generated.

## Interrogation of LMS physiology—function, structure and biochemistry

**Fig. 2:**
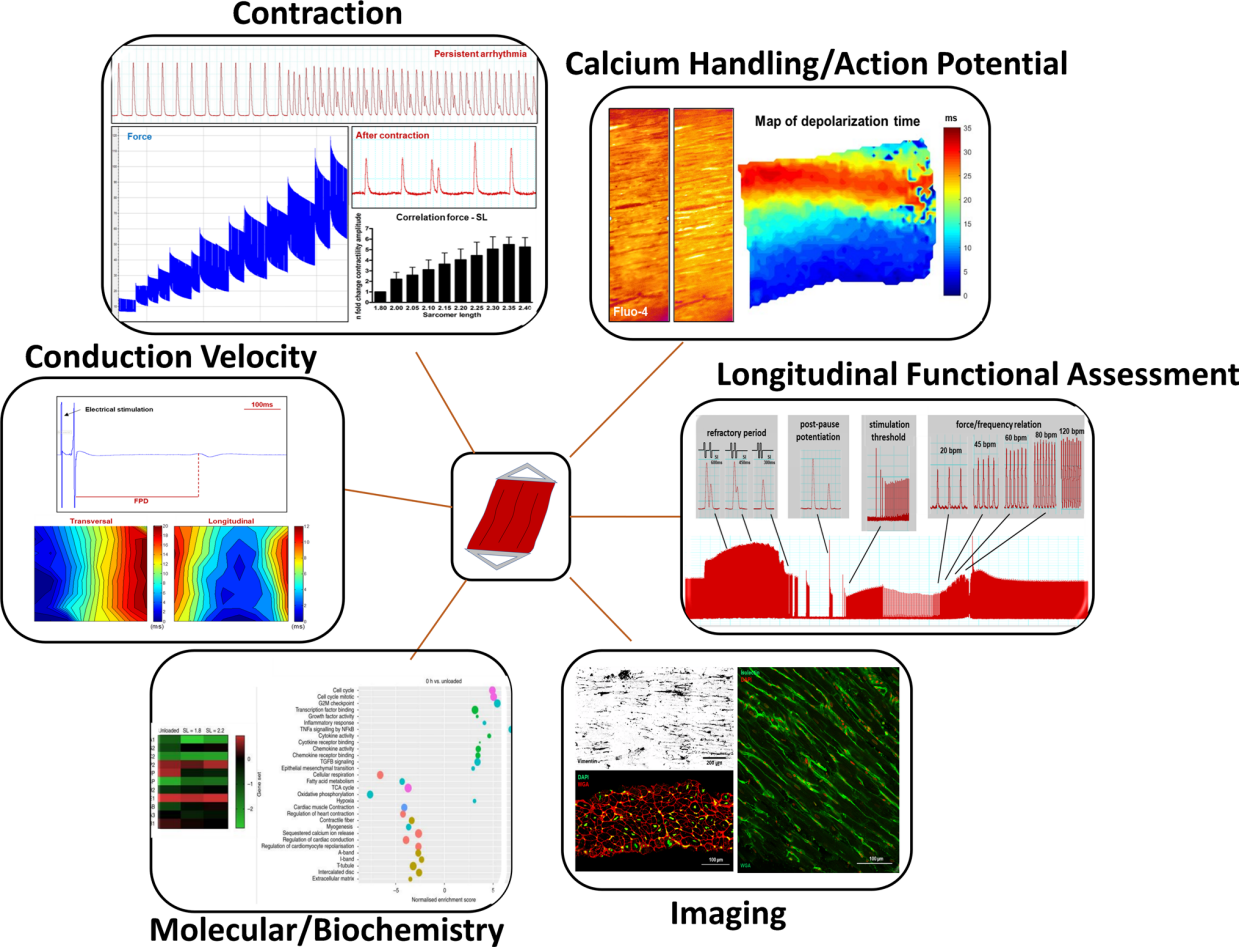
Interrogation of LMS function, structure and biochemistry. Several assays can be used to interrogate the function, structure and biochemistry of myocardial slices. Functional assessment includes contractile function, conduction velocity, Ca^2+^ handling, action potential, metabolomics and viability. Structural assessment can be conducted using traditional sectioning and staining methods. Additionally, immunohistochemical staining and microscopy can easily be performed and optical clearing protocols have been developed for whole slice imaging. Biochemistry assays can be conducted at the genomic, proteomic and secretomic levels. Longitudinal functional assessment of LMS can also be performed during chronic culture. The RNA sequencing image in this figure was previously published [43]

A large variety of assays can be applied to interrogate LMS function, structure and biochemistry (Fig. 2). Functional assays include force measurements, conduction velocity, Ca^2+^ transients, action potential, metabolism and viability. For such measurements, LMS should be given some time to recover from the cold temperatures required for slicing, wash out of the excitation–contraction uncoupler, and the re-activation of the contractile apparatus. Wang et al. demonstrated a clear effect of temperature as the main contributor for an initial and transient alteration of action potentials [42]. Structural assessment provides analysis of cellular and ECM organization, and specific biomolecules can easily be labeled and visualized. Biochemical assessment can be used to assess the genomic and proteomic signatures of LMS. Several of these assays can be robustly reproduced, with no need for further characterization or optimization [36, 43, 44]. However, it is important to understand how LMS features are characterized and how these data may be integrated with and utilized for other applications and to address the questions of several research fields.

## Contractile function

Contraction is the primary function of the myocardium. Cardiac remodeling and changes in mechanical load affect contractile function, with corresponding changes at the structural and molecular level [37]. Contractile function measurements are typically performed in an organ bath employing an isometric force transducer. Electrical stimulation is applied either by point stimulation (by using a microelectrode) or field stimulation (platinum or graphite electrodes). Adaptors (various shapes and materials) are attached by cyanoacrylate glue to the edges of the myocardial slice perpendicularly to the myocardial fiber orientation and LMS are progressively stretched. Developed forces should be normalized to muscular cross-sectional area and reported as wall stress (mN/mm^2^). Like other cardiac preparations, the Frank–Starling relationship can be observed in LMS (Fig. 2) [17, 36]. Reported contractility parameters (isometric force measurements) indicate that freshly prepared viable myocardial slices should generate a force of ~ 15 mN/mm^2^ [43, 44]. Contraction and relaxation kinetics (time to peak, time to 50% relaxation (t50), t90, decay rate and raise time/rise slope) can be investigate alongside force measurements, which may provide additional functional insight. Recent findings from Pitoulis et al*.* have demonstrated transmural differences in contractile function, where LMS from the epicardium and mid-myocardium had a higher active and passive tension than those generated from the endocardium [36]. This aspect is often overlooked when using isolated cardiomyocytes, and something that should be considered during experimental design.

Previous publications have demonstrated how prolonged in vitro culture affects LMS contraction [9, 43]. Recent technological developments by Fischer et al. have allowed constant monitoring of tissue contraction during in vitro culture [9]. Stimulation threshold, tonic tissue contracture, arrhythmic events and changes to the force–frequency relationship, which often occur during in vitro culture or pharmacological stimulation [1], can also be easily acquired. Additionally, several pacing strategies have been developed to assess long-term contractile function such as refractory period and post-pause potentiation (Fig. 2). The longitudinal assessment of these parameters could be used to investigate how culture duration and drug treatments modulate LMS functional properties [9].

Contractile function has also been used to assess effects of conductive tissue patches, indicating this assay can be an alternative to ex vivo Langendorff heart measurements [22, 27]. Additionally, the contractile response can be assessed after pharmacological stimulation, and isoproterenol stimulation studies have been used to assess the potential for arrhythmia in cultured LMS [43].

Small muscle strips (300–400 µm wide) can also be dissected from LMS and used to measure tissue contraction. This approach was used by Thomas et al*.* to demonstrate the effect of Alpha-1A-adrenergic receptor signaling in the human heart [28]. New methods to measure contraction are currently being developed. In a recent study by Schubert et al*.*, micro- and nanoscopic lasers were used for optical recording of transient cardiac contraction profiles [39]. These recordings were not restricted to the tissue surface, but could sense though hundreds of micrometers of rat heart tissue. This approach opens new frontiers for non-invasive monitoring of a wide range of physiological parameters with cellular resolution.

## Conduction studies

Conduction velocity (CV) is the speed at which an electrochemical impulse propagates through cardiac tissue. A wide array of factors, including age, sex and cardiac tissue remodeling can affect conduction velocity [16, 24, 40]. In the laboratory setting, assessment of CV is often performed with multi-electrode arrays and this methodology is easily applied to LMS by placing the preparation onto the array and immobilizing it with a net or a weight to ensure good contact between the tissue and electrodes. Highly functional LMS generate a clear signal where the spread of activation is fastest in the direction of the myocardial fibers (longitudinal) and slow when transverse to fiber direction (transversal) [25]. The analysis of these parameters is conducted off-line, after laboratory experimentation, and it is based on the “single vector” or “average” method which has been extensively described [25] and implemented by computerized analysis [19]. Field potential duration, which is analogous to action potential duration [13], can also be studied in this setup (Fig. 2). Fragmented signals are an indication of a damaged or deteriorating LMS. During experiments, manipulation of LMS should be kept to a minimum and LMS should be left to stabilize for a few minutes prior to initiation of data collection. It is also advised not to trim LMS as this can mechanically trigger arrhythmic activity. Current published methods could be further improved by the use of flexible electrodes on physiologically loaded and contracting LMS, which are likely to further improve the quality of data collected.

## Ca^2+^ handling and action potential analysis

Analysis of action potentials and Ca^2+^ transients provides further mechanistic insight into cardiac tissue contraction in health and disease. Optical mapping, a standard technique used to acquire fluorescence images, can be applied to LMS to provide insight into transmembrane potential, intracellular [Ca^2^ +], as well as other critical physiological parameters. Application of non-ratiometric probes, such as Fluo-4 AM or Fluo-8 AM, has already been reported for the assessment of intracellular Ca^2+^ dynamics [20, 21, 42, 43]. Some examples of popular voltage-sensitive dyes used to record action potentials (AP) are Di-4-Anneps, Di-8-Anneps, RH237 [15, 21, 42]. The use of an upright microscope facilitates data acquisition directly from the LMS surface. The presence of an uneven surface can be easily overcome by re-adjusting the focus. If an inverted microscope is used, the use of a glass coverslip increases the acquisition distance which can result in light scattering and lower resolution. This technical limitation can be overcome using long working distance lens.

Some of the most commonly used parameters to describe calcium kinetics are Ca^2+^ transient amplitude, time to peak, rise slope, time to 50% decay, time to 90% decay and Ca^2+^ decay rate. Analysis of action potential dynamics can also be conducted in the same manner. Single cardiomyocytes or large areas which include few (30–50 cells) to hundreds or thousands of cells can be assessed. When conducting these experiments, the indicator loading protocol has been reported to change according to the species of tissue used. Smaller mammal LMS, such as rat/mice, require shorter probe loading and de-esterification times, than larger mammal LMS, such as human [44]. Sufficient time for the LMS to recover from the slicing process should be given to ensure the LMS to reach steady-state electrophysiological properties and thus produce reliable AP recordings [42]. To enhance LMS imaging, the indicator should be loaded while the LMS is physiologically loaded and electrically stimulated at 37 °C, maintaining LMS in a functionally active and physiological state. Attention should also be dedicated to the loading of fluorescence probes as, particularly in the case of voltage dyes, indicator overload results in tissue toxicity and cell death. Pluronic F-127 can be added to the indicator to stabilize the cardiomyocyte cell membrane and improve indicator retention. When non-ratiometric calcium indicators are used, myosin inhibitors (such as Blebbistatin) or 2,3-butanedione monoxime (an excitation–contraction uncoupler) must be added to minimize movement artifact during data acquisition. In the latter case, the effects of 2,3-butanedione monoxide on the electrical restitution of cardiomyocytes [23] should be taken into consideration. Challenges of data analysis and interpretation should also be considered and we redirect the reader to the article by Wang et al. for a more detailed discussion [42]. The fluorescent signal generated by LMS is often weaker than those of other multicellular preparations, thus multiple routines to increase the reliability of the data, which reduces the impact of noise distortion, have to be included. Multiple-site point stimulation and field stimulation are recommended to uncover electrophysiological tissue heterogeneity associated with variable cell alignment and to exclude contributions of source–sink mismatches. Optical mapping also generates large amounts of data for which an automatic or semi-automatic analysis is recommended [42]. A powerful development would be the use of ratiometric dyes and associated imaging systems. This approach relies on two fluorescence intensities and it allows correction of artifacts due to bleaching or to changes in focus induced by tissue contraction. Although data processing is more complicated, this method has been used in other multicellular cardiac preparations for the simultaneous recording of force, calcium and action potential parameters [25]. The adaptation of this approach would overcome several flaws of non-ratiometric dyes and could be used to answer more specific questions related to cardiac tissue contraction.

## Metabolomics and mitochondria

Myocardial bioenergetics are an established regulator of tissue remodeling and cellular dedifferentiation [12], as well as a major target for the induction of cardiomyocyte maturation [18]. Several research techniques have been developed to investigate both cardiac metabolism and mitochondrial activity. Most of them are designed for whole heart preparations or in vitro cell monolayers. Custom-made adaptations are often required for these techniques to be used on LMS, which have significantly limited their utilization. Mitochondria can easily be labeled with Mitotracker™, which binds to mitochondrial proteins regardless of mitochondrial membrane potential. Tetramethylrhodamine methyl ester (TMRM) is a dye that is readily sequestered by mitochondria and can be used to assess membrane potential in real time, and hence the effects of drugs/stimuli. During image acquisition, physiological conditions such as electrical stimulation and mechanical load are recommended. Alternatively, mitochondria have been investigated using immunohistochemical staining and confocal microscopy, following paraformaldehyde fixation (discussed in *Structural Parameters*). Extensively used methods for real-time measurements of oxygen consumption such as Clark-type oxygen electrode could be performed on LMS [8], but such experiments have yet not been reported in literature. Additionally, carbonyl cyanide *p*-trifluoromethoxyphenylhydrazone (FCCP) and oligomycin can be added to measure maximal rates of oxygen consumption and ATP synthase-independent proton leak, respectively. Alternatively, commercially available Live-cell Metabolic Assay Platform could be optimized to investigate oxygen consumption and extracellular acidification rate to interrogate key cellular functions such as mitochondrial respiration and glycolysis. The thinness of LMS results in almost instantaneous preservation of molecular integrity following snap freezing. As such, accurate quantification of metabolite concentrations, including ATP, ADP, AMP, phosphocreatine, creatine, NAD and NADH, can be achieved using high performance liquid chromatography [4, 43]. The diversity of techniques available to interrogate cardiac metabolism within LMS could be applied to animal models of metabolic cardiovascular disease, such as diabetes, providing new avenues for further understanding and therapeutic intervention.

## Viability

Viability of LMS has been mainly assessed using two approaches: (1) LIVE/DEAD staining, or (2) metabolic assays. LIVE/DEAD staining provides a two-color fluorescence assessment that is based on the simultaneous determination of live and dead cells with probes that recognise ubiquitous intracellular esterase activity (calcein AM) and plasma membrane integrity (ethidium homodimer-1). Automated imaging of the entire LMS surface can be achieved using a widefield microscope equipped with a motorized stage [43]. Alternatively, higher resolution assessment can be conducted acquiring images of the slice surface using a fluorescence or confocal microscope [44]. The use of higher magnification objectives may be more informative when specific areas, such as infarct border zones, are investigated. This approach is limited to the assessment of the slice surface viability, as neither a confocal nor widefield microscopes are able to image more than two to three cell layers deep. The quantification is therefore based on a mathematical calculation which is based on optical quantifications [28, 33]. These findings should therefore be further validated with alternative approaches. The quantification of whole slice viability is also assessed using metabolic assays, such as CellTiter96 AQ_ueous_ One Solution Cell Proliferation assay [43], MTT assay [29] or alamarBLUE cell viability reagent. Initial optimization experiments should be performed to determine optimal incubation time. These approaches are very fast and can easily be applied to simultaneous analysis of several treatments or conditions. For these reasons, they are often preferred to contractile function, conduction velocity or optical mapping measurements where sampling is restricted. The limitation of such methods is that LMS cannot be electrically stimulated during the assay and must be removed and trimmed from the culture apparatus, inducing LMS deterioration. Micro-incubation chambers capable of maintaining the tissue mechano-electrically stimulated are likely to further enhance the sensitivity and reliability of these assays. Another important aspect to consider for in vitro cultured LMS is the influence of other cardiac cell types. During in vitro culture, cardiomyocytes remodel and reduce their metabolic activity, while cardiac fibroblasts are activated and proliferate [3, 33]. These changes will ultimately confound the viability readings as preserved metabolic activity would not correspond to preserved tissue function.

## LMS structure: visualizing cells, ECM and biomolecules

**Fig. 3:**
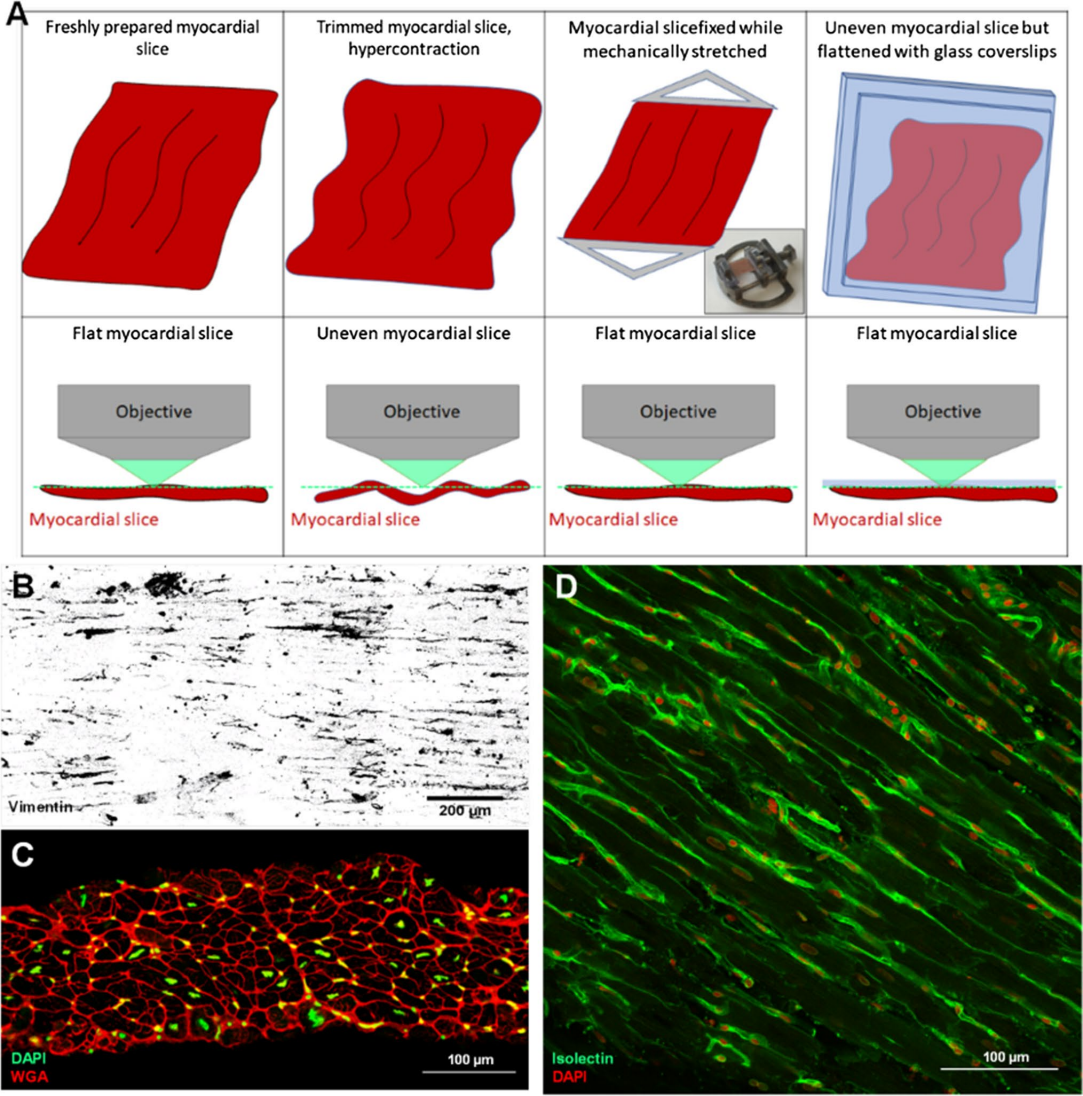
Imaging LMS. **a** Imaging of LMS is best conducted when LMS are flat, allowing acquisition of images of cells within a single plane. During fixation, cardiomyocyte hypercontraction can result in tissue deformation and folding, which makes image acquisition time consuming and technically challenging. This can be overcome by performing fixation with the LMS mechanically stretched and imaging performed, while the slice is flattened with a glass coverslip. **b** Representative vimentin staining of rat LMS. **c** Cross section of LMS stained for WGA (in red) and DAPI (in green). **d** LMS stained for isolectin-B4 (in green) and DAPI (in red) to identify endothelial cells and cardiac cell nuclei

LMS facilitate the investigation of cardiac structure within an intact and minimally disrupted in vitro environment. Standard paraffin or optimum cutting temperature (OCT) compound processing methods can be used to generate longitudinal or transverse microsections [3, 28]. To minimize structural alterations that may occur during tissue processing (freezing or cutting), samples can be fixed with 4% PFA (or other fixation methods) and directly used for imaging. Standard immunostaining protocols can be used, although incubation steps should be lengthened and/or include mechanical agitation to encourage adequate diffusion of the reagents into the fixed myocardial slice. *Z*-series of images can be acquired and combined to obtain a final high-quality 3D reconstructed representation of the myocardial slice surface. Various imaging methods can then be applied to visualize large or extremely small portions of the samples, thus investigating targets at the multicellular, cellular or subcellular level (Fig. 3b). Previous publications have shown that informative data such as cardiomyocyte morphology, t-tubules or mitochondria density and regularity, and stromal cell numbers can be acquired [1, 32, 43]. Heterogeneity in cardiomyocyte morphology and particularly to their aspect ratio (length:width) has recently been demonstrated in LMS generated from the epicardium compared to midmyocardium and endocardium [36]. Subcellular targets such as connexins (density and distribution) can be identified and subsequently correlated with conduction velocity parameters [43]. Given the high density and the opaque nature of the myocardium, deep three-dimensional (3D) imaging is difficult to achieve using conventional approaches. Optical clearing techniques are an option to circumvent this [32]. They can be a crucial tool for 3D investigation of the cellular and tissue-level organization, providing comprehensive investigation of the structure of cardiovascular tissue, which can then be related to whole heart data.

## Biochemical analysis

Biochemical analyses are normally conducted at the genomic, proteomic and secretomic levels and related methods can be easily applied to LMS. For LMS, as well as for other cardiac models, snap freezing in liquid N_2_ is recommended for the optimal preservation of genetic material and proteins. At this point, the tissue slices are no longer alive; therefore, they will be referred to as myocardial slices (MS). Isolation of genetic material from MS is performed following standard procedures indicated for cardiac tissue/multicellular preparations [17]. Following disruption of the frozen MS sample, off the shelf kits can be used to isolate various RNA types, including abundant mRNAs as well as less expressed long non-coding RNAs or microRNAs. Specific RNA transcripts can then be quantified using qPCR; alternatively, RNA sequencing can be used to obtain the total transcriptomic profile. While it provides huge amounts of data from all of the cell types within the MS, RNA sequencing is still expensive and analysis is often challenging. Novel integrative bioinformatics pipelines are now being developed to decipher essential biological insights from global transcriptome profiling data. Fuchs et al. recently applied this concept to LMS treated with anti-fibrotic compounds, resulting in the identification of two microRNAs linked to fibroblast signaling via the analysis of altered gene sets associated with fibroblast proliferation, ECM remodeling and wound healing [10] With regard to the protein content of MS, specific proteins can be detected and extracted using traditional techniques, including Western blot [28, 36]. More detailed studies of the proteomic composition of LMS have been conducted. Mass- spectrometry-based proteomic techniques, such as shotgun and SRM (selective reaction monitoring) can be complemented with SWATH-MS, a data independent acquisition method that combines the high throughput of the shotgun method and the high reproducibility of the SRM method. Like RNA sequencing, proteomic analysis is still challenging and current analytical techniques are not able to fully integrate the data obtained from both. Powerful computational techniques are now required to fully interrogate and integrate the data that can be harvested, which will be an important next step in the cardiovascular science field. Alongside the interrogation of MS intracellular biochemistry, analysis of spent medium can be conducted. Data on the secretome, exosomes and microRNAs can be obtained, although this experimentation has not yet been reported in the literature. One issue is the relatively small number of cells within LMS, compared to the millions of cells that are typically used for these in vitro evaluations. Additionally, some of the LMS in vitro culture methods [43] require relatively large volumes of media, resulting in low concentrations of secreted molecules. Novel in vitro culture techniques are reducing the volume of medium required [9], making it likely these analyses will become increasingly popular.

## Isolating cells from LMS

**Fig. 4:**
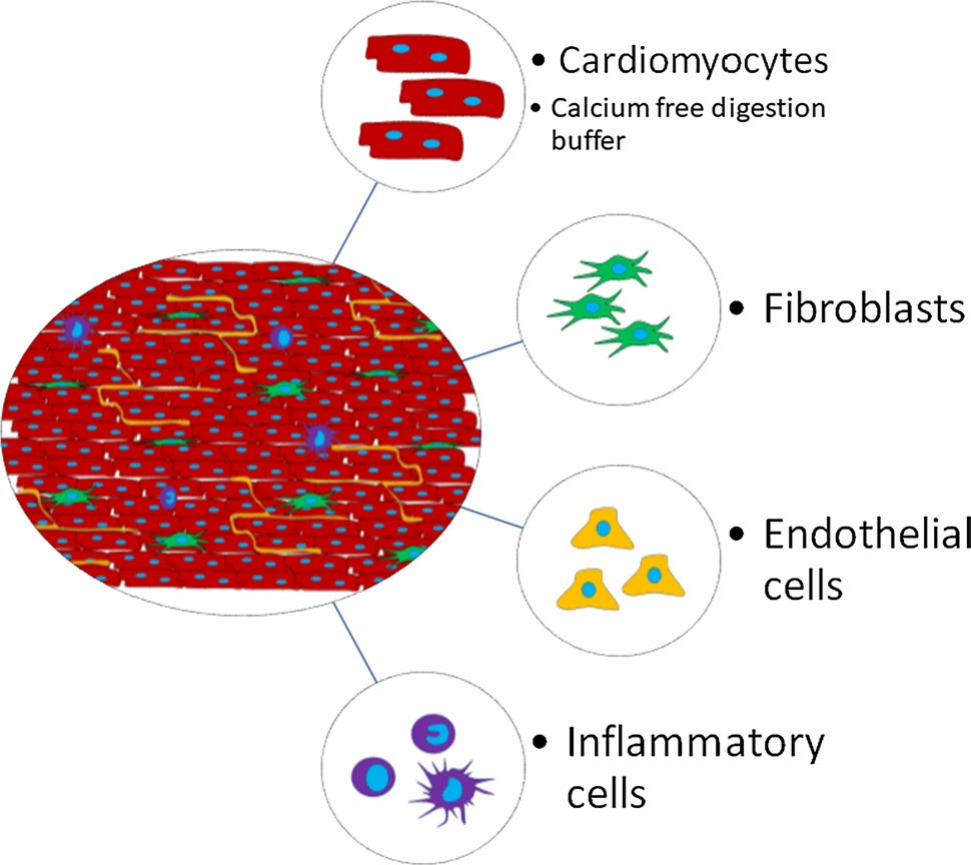
Isolating cells from LMS. LMS can be broken down into constituent cell populations, including isolation of cardiomyocytes, fibroblasts, endothelial cells, inflammatory cells or any other cardiac cell type

Specific cell populations can be harvested from freshly prepared or cultured LMS for single cell investigations as a means of propagation or to carry out molecular or cellular analyses (Fig. 4). Cell-specific RNA and protein can then be extracted and processed [43]. Techniques used on tissue samples, including mechanical and/or enzymatic disruption, can be useful for liberating the cells but must be performed with great care to maintain the integrity of both surface or intracellular components while still generating sufficient yields. This is particularly important when flow cytometry analysis is required. Specific protocols should also be utilized according to the cell type of interest [17, 26, 35]. For example, calcium-free digestion buffers are recommended for cardiomyocyte isolation [17], whereas they are not needed for stromal cells such as endothelial cells or fibroblasts. A recent study by Fiegle et al*.* has demonstrated that isolation of excitable and contractile cardiomyocytes is more efficient from LMS than from tissue chunks and results in a higher yield in both rat and human specimens [7].

## Future perspectives

The publication of an optimized and reproducible protocol [44] to generate highly viable and functional LMS has broadened the interest and utilization of this technique among academic and commercial laboratories. The initial stages of development and validation have now been surpassed and expected results for several assays are available. Further refinement in the methodology as well as education and validation of research readouts is needed to ensure experimental reproducibility between laboratories. Following a broad LMS characterization, current research is likely to focus on a deeper understanding of specific aspects of the myocardium, such as regional or transmural differences, as well as species-dependent differences and pathological changes associated with particular disease states. For the latter, a better LMS characterization prepared from various human pathological conditions as well as from pathological animal models is warranted. The possibility of prolonged tissue storage has already broadened the diversity of samples that can be utilized, while reducing time pressure associated with the use of fresh tissue. It will also allow tissue samples to be transported and shared between research groups, fostering the formation of strong collaborations and networks between research units. Various groups, with individual specialist expertise, will be able to work on the same samples, thus acquiring, in parallel, a larger number of integrated datasets. Future efforts should also be dedicated to the development of cryopreservation methods, as LMS thinness should facilitate a homogenous freezing process throughout the preparation^21,26^.

LMS thinness is a clear advantage over the other cardiac multicellular models as it allows the diffusion of oxygen and nutrients, thus preventing ischemic damage and preserving viability. For this reason, scientists have tried various strategies to maintain LMS in vitro [3, 30, 41]. New biomimetic approaches have recently demonstrated that LMS can be maintained in a highly functional state for prolonged periods in vitro, further expanding the interest in LMS use. By replicating some of the in vivo environment, with humoral, electrical and mechanical stimulation, biomimetic technologies can delay tissue remodeling and cellular dedifferentiation [9, 29, 43]. However, it is important to notice that tissue adaptation is still occurring and this should be taken into consideration for chronic in vitro studies. Next-generation RNA sequencing indicated that a large number of genes sets are altered within 24 h of in vitro culture, indicating a fast response of cardiac cells to the new artificial environment [44]. It has also been observed that following the initial acclimatization to in vitro conditions, LMS seem to adapt to the in vitro environment and reach a stable state. This adaptation could potentially be controlled by culture conditions and eventually level out variability among diseased samples. Although it is unlikely that this adaptive process can be prevented, the significant advances made clearly indicate that improved media formulations as well as more sophisticated humoral and mechanical stimulations will be essential to further delay, or manipulate, this process. The standardization of biomimetic conditions and technologies for long-term cultivation will also facilitate inter-laboratory experimental reproducibility. These developments are and will be essential for representative and reliable chronic in vitro experiments and can be applied to traditional pharmacological and hormonal stimulation studies, which, until now, could only be reliably conducted at acute time points. On this topic, the response of in vitro cultured LMS to pharmacological interventions or cardiac toxicity protocols should be compared to alternative high-throughput platforms, such as iPSC-derived myocytes or engineered heart tissue. Being a native adult multicellular preparation, LMS could become a powerful asset for translational research and drug development.

As a proof of concept, myocardial slices have been effectively transfected with viruses [28, 29]; however, the prolonged preservation of LMS viability and functionality provided by novel biomimetic approaches will give a new dimension to these studies. Novel molecular tools, such as CRISPR-Cas9, locked nucleic acids and viral-mediated gene transfer, will allow us to genetically manipulate cardiac tissue and acquire essential information to broaden both therapeutic and mechanistic understanding. Furthermore, single cell type-specific promoters [17] can facilitate the targeting, evaluation and modulation of specific cardiac cell types. Finally, LMS offer a new and exciting opportunity to investigate cell integration, maturation and electrotonic interactions between grafted cells and the native myocardium [14] for the purpose of cell therapy. The visualization of such processes at a cellular/subcellular level is particularly challenging in other organotypic model systems.

To conclude, the myocardial slice model is being accepted on the main stage of cardiovascular research and the interest in the technology is certainly growing as demonstrated by the increasing number of recent publications. For now, it is important to maintain an open, collaborative and multidisciplinary approach to tackle current challenges in cardiovascular research. Recent developments have already opened new frontiers for translational research and further mechanistic understanding of cardiac (patho-)physiology will soon translate into innovative therapeutic strategies.
